# The truncated somatostatin receptor sst5TMD4 stimulates the angiogenic process and is associated to lymphatic metastasis and disease-free survival in breast cancer patients

**DOI:** 10.18632/oncotarget.11076

**Published:** 2016-08-05

**Authors:** Manuel D. Gahete, David Rincón-Fernández, Mario Durán-Prado, Marta Hergueta-Redondo, Alejandro Ibáñez-Costa, Alejandro Rojo-Sebastián, Francisco Gracia-Navarro, Michael D. Culler, Oriol Casanovas, Gema Moreno-Bueno, Raúl M. Luque, Justo P. Castaño

**Affiliations:** ^1^ Maimonides Institute for Biomedical Research of Cordoba (IMIBIC), Cordoba, Spain; ^2^ Department of Cell Biology, Physiology and Immunology, University of Cordoba, Cordoba, Spain; ^3^ Reina Sofia University Hospital (HURS), Cordoba, Spain; ^4^ CIBER Physiopathology of Obesity and Nutrition (CIBERobn), Cordoba, Spain; ^5^ Department of Biochemistry, Universidad Autónoma de Madrid (UAM), Instituto de Investigaciones Biomédicas “Alberto Sols” (CSIC-UAM), IdiPAZ, MD Anderson Internacional Foundation, Madrid, Spain; ^6^ Pathology Deparment, MD Anderson Cancer Center, Madrid, Spain; ^7^ IPSEN Bioscience, Cambridge, Massachusetts, USA; ^8^ Tumor Angiogenesis Group, Catalan Institute of Oncology-IDIBELL, L'Hospitalet de Llobregat, Barcelona, Spain

**Keywords:** somatostatin receptor, sst5TMD4, breast cancer, angiogenesis, VEGF

## Abstract

The truncated somatostatin receptor sst5TMD4 is associated with poor prognosis in breast cancer and increases breast cancer cell malignancy. Here, we examined the cellular/molecular mechanisms underlying this association, aiming to identify new molecular tools to improve diagnosis, prognosis or therapy. A gene expression array comparing sst5TMD4 stably-transfected MCF-7 cells and their controls (empty-plasmid) revealed the existence of profound alterations in the expression of genes involved in key tumoral processes, such as cell survival or angiogenesis. Moreover, sst5TMD4-overexpressing MCF-7 and MDA-MB-231 cells demonstrated increased expression/production of pro-angiogenic factors and enhanced capacity to form mammospheres. Consistently, sst5TMD4-expressing MCF-7 cells induced xenografted tumors with higher VEGF levels and elevated number of blood vessels. Importantly, sst5TMD4 was expressed in a subset of breast cancers, where it correlated with angiogenic markers, lymphatic metastasis, and reduced disease-free survival. These results, coupled to our previous data, support a relevant role of sst5TMD4 in the angiogenic process and reinforce the role of sst5TMD4 in breast cancer malignancy and metastatic potential, supporting its possible utility to develop new molecular biomarkers and drug therapies for these tumors.

## INTRODUCTION

Cancer represents one of the most serious and complex health threats worldwide [1]. In particular, breast cancer is the most common tumor type in women and the second leading cause of death after lung carcinoma [1]. Unfortunately, the extraordinary variability, heterogeneity, and complexity of cancer, hampers the finding of common molecular elements, which could facilitate the development of more general and effective diagnostic and therapeutic strategies. In this regard, it has been established during the last years that most cancers share a group of common hallmarks such as sustained proliferative signaling, evasion of growth suppressors, resistance to cell death, replicative immortality, angiogenesis, activation of invasion and metastasis, genome instability, inflammation, altered energy metabolism and evasion to immune destruction [2, 3]. More recently, it has been suggested the existence of additional common hallmark shared by all the tumoral pathologies as is the case of altered alternative splicing processes [4]. Indeed, recent studies point out that intratumoral heterogeneity in outcome and cancer survival can be explained, at least in part, by genetic variations (such as splicing variants) present in the primary tumor [5].

In line with this, our group has recently identified a new truncated variant of the somatostatin receptor subtype 5, sst5TMD4, which is derived from a non-canonical splicing process [6], and is associated with the progression and/or malignancy of several endocrine-related tumors [6–12]. This truncated receptor was initially identified and characterized in pituitary adenomas [6], where its presence is associated to more aggressive features [8], and a poorer response to the classical medical therapy in this pathology, the somatostatin analogues [12]. Similarly, sst5TMD4 was also found to be overexpressed in poorly differentiated thyroid cancers, where it may explain the lack of response to somatostatin analogue treatment [9]. Most notably, sst5TMD4 presence was correlated with a worse prognosis in a group of breast cancers, and its overexpression was associated with increased malignant features in cell lines derived from breast tumors (MCF-7) [10]. Taken together, these results demonstrate a relevant role of the truncated receptor sst5TMD4 in several endocrine-related tumor pathologies wherein somatostatin or its analogs could be playing a regulatory role [11].

Based on these previous results, this study was aimed to attain a further understanding of the cellular and molecular mechanisms underlying the association between sst5TMD4 and bad prognosis and malignancy in breast cancer, with the ultimate goal of identifying new molecular targets with potential utility as biomarkers to improve diagnosis, predict prognosis or develop novel therapies in these tumors. To pursue this goal, we determined the gene expression microarray profiling of sst5TMD4-overexpressing MCF-7 cells, which, after functional enrichment analysis, revealed that several modified genes were tightly related, among other biological functions, to angiogenic process. Accordingly, we employed several relevant models, including *in vitro* cell lines, xenografted tumors and human breast cancer samples, to explore the association between the presence of the truncated receptor sst5TMD4 and the angiogenic process, as well as the possible relationship between this truncated receptor and relevant clinical features of the breast cancer patients.

## RESULTS

### sst5TMD4 overexpression alters the expression of angiogenesis-related genes in MCF-7 cells

**Figure 1:**
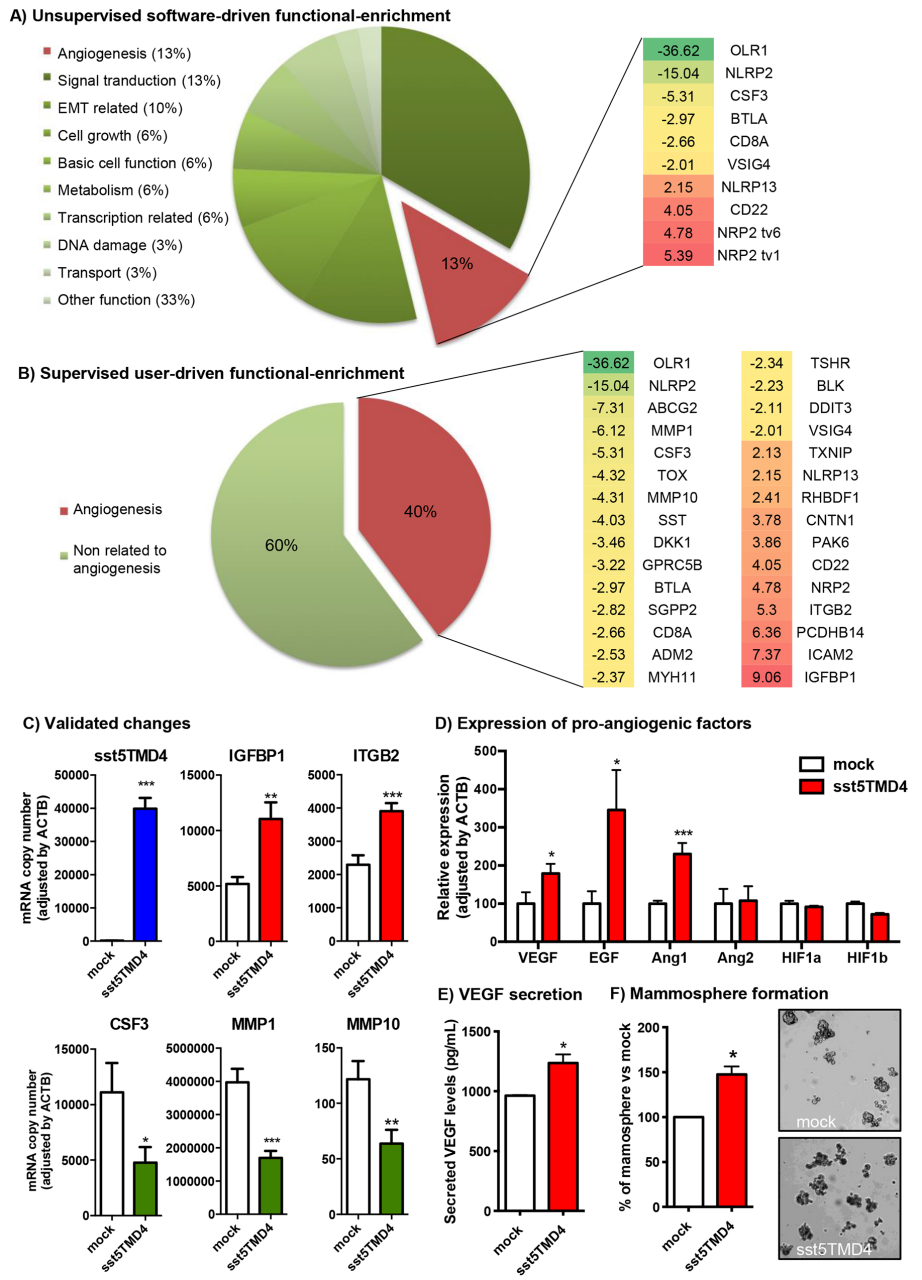
sst5TMD4 expression is associated to higher expression of pro-angiogenic factors and higher capacity to form mammospheres in breast cancer MCF-7 cells **A.** Software-driven functional analysis of genes whose expression is altered by the presence of sst5TMD4 in MCF-7 cells by gene expression microarray (green = inhibition, red = overexpression). **B.** User-driven supervised functional analysis of genes whose expression is altered by the presence of sst5TMD4 in MCF-7 cells by gene expression microarray (green = inhibition, red = overexpression). **C.** Examples of sst5TMD4-induced gene expression changes validated by additional qPCR in transfected cell lines. **D.** Changes in the expression of angiogenesis-related genes (VEGF, EGF, Ang1, Ang2, HIF1a and HIF1b) measured by qPCR in MCF-7 cells stably transfected with sst5TMD4 or pCDNA3.1 empty vector (mock). **E.** Levels of secreted VEGF in MCF-7 cells stably transfected with sst5TMD4 and mock controls measured by ELISA. **F.** Percentage and representative images of mammospheres formed from MCF-7 cells stably transfected with sst5TMD4 and the respective mock controls. Data represent mean ± SEM of n=3-6 independent experiments. Asterisks (*, p<0.05; **, p<0.01; ***, p<0.001) indicate significant differences between sst5TMD4- and mock-transfected MCF-7 cells.

A gene expression microarray comparing sst5TMD4-transfected MCF-7 cells with mock-transfected MCF-7 cells revealed an elevated number of genes altered by sst5TMD4 overexpression (38% up-regulated and 62% down-regulated) (Supplementary Table S1). Indeed, a software-driven functional-enrichment analysis of these data indicated that sst5TMD4 overexpression in MCF-7 alters the expression of numerous genes involved in relevant cellular processes such as epithelial to mesenchymal transition (EMT) (10% of altered genes with known function), cell growth (6%), cell metabolism (6%), signal transduction (13%), or, notably, angiogenesis (13%) (Figure 1A). Consistent with this analysis, it was previously demonstrated that sst5TMD4 overexpression could alter cell growth and EMT, among other tumor-associated processes [10]. Conversely, its putative implication in the angiogenic process has not been explored hitherto. In support of the interest of exploring this issue, a user-driven functional-enrichment analysis of the data generated revealed that 31 out of 78 genes with known function (40%) significantly altered by the presence of sst5TMD4 were associated to the angiogenic process, based on the previous literature (Figure 1B and Supplementary Table S1). Further validation, by qPCR, of the genes that were altered in the gene expression microarray, confirmed the regulation of several angiogenesis-related genes in sst5TMD4-overexpressing MCF-7 cells, including the overexpression of ITGB2 or IGFBP1 (Figure 1C).

### sst5TMD4 increases the production of pro-angiogenic factors and the capacity to form mammospheres in MCF-7 and MDA-MB-231 cells

In that the angiogenic process is driven by the expression and secretion of pro-angiogenic factors, the expression of the most relevant growth factors with angiogenic activity was determined in sst5TMD4-expressing cells. In particular, sst5TMD4-transfected cells showed elevated levels of VEGF (p<0.05), EGF (p<0.05) and Ang1 (p<0.001), with no changes in Ang2 expression, compared with mock-transfected cells (Figure 1D). However, this elevation in the expression of pro-angiogenic factors was not accompanied by an increase in the expression of hypoxia-induced genes such as HIF-1a or HIF-1b (Figure 1D), whose elevation generally precedes those of the pro-angiogenic factors. Nevertheless, changes in protein expression or phosphorylation of these proteins cannot be ruled out. Consistent with these results, ELISA measurements revealed higher levels of secreted VEGF in culture medium from sst5TMD4-transfected cells compared to mock controls (Figure 1E), supporting the implication of sst5TMD4 in the elevated expression and secretion of angiogenic factors in MCF-7 cells. Since angiogenesis in tumors seems to significantly derive from cancer stem-like cells (CSC)-secreted pro-angiogenic factors [13–15], we aimed to determine the capacity of sst5TMD4-overexpressing MCF-7 cells to form mammospheres in low-adherence surfaces. This demonstrated that the presence of sst5TMD4 not only enhances the production of pro-angiogenic factors, but also increases the population of CSCs (Figure 1F), suggesting a role in maintaining the population of CSCs in breast cancer. Remarkably, the most relevant changes observed in MCF-7 were reproduced in an additional breast cancer cell model, the MDA-MB-231 cell line. Indeed, sst5TMD4-overexpressing MDA-MB-231 cells showed significantly elevated levels of two of the main pro-angiogenic factors (VEGF and EGF) and higher capacity to form mammospheres (Supplementary Figure S1), confirming the role of the truncated sst5TMD4 receptors in the angiogenic process.

### sst5TMD4 increases VEGF production and angiogenic features in an *in vivo* xenograft model

**Figure 2:**
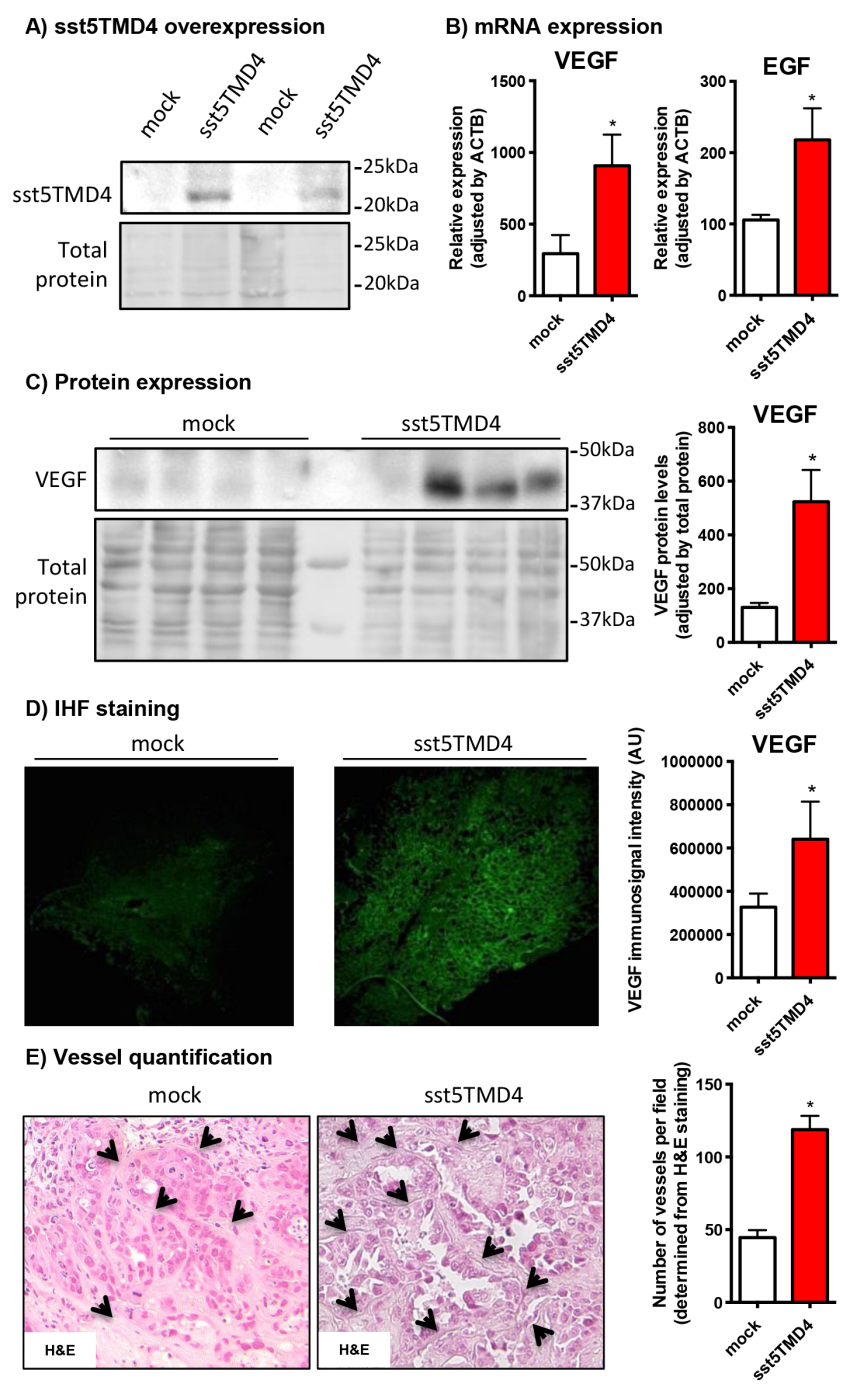
sst5TMD4 overexpression increases the expression of pro-angiogenic factors and the number of blood vessels in xenografted breast cancer cell line-derived tumors **A.** sst5TMD4 protein expression by western blotting in mock- and sst5TMD4-MCF-7 xenografted tumors. **B.** Expression levels of EGF and VEGF in xenografts derived from mock- and sst5TMD4-MCF-7 cells measured by qPCR. **C.** and **D.** VEGF protein expression by western blotting and IHF in mock- and sst5TMD4-MCF-7 xenografted tumors. **E.** Representative images (x20) and quantification of straight blood vessels in xenografts derived from mock- and sst5TMD4-MCF-7 cells. Data represent mean ± SEM of n=4-5 samples. Asterisks (*, p<0.05) indicate significant differences between sst5TMD4- and mock-transfected tumors.

In order to confirm a relevant association between the expression of the truncated receptor and the angiogenic process in a preclinical model, we analyzed the expression of pro-angiogenic factors in xenograft tumors generated by the inoculation of sst5TMD4-overexpressing MCF-7 cells in nude mice, as compared to mock transfected cells [10]. Consistent with the results obtained *in vitro*, these *in vivo* xenografted tumors induced by sst5TMD4-transfected cells (Figure 2A) showed elevated VEGF and EGF expression levels (by qPCR; Figure 2B) and increased VEGF protein (detected by western-blot and immunohistofluorescence), as compared to tumors generated by the inoculation of mock-transfected cells (Figure 2B-2D). In addition, according with these results, tumors induced by sst5TMD4-overexpressing MCF-7 cells exhibited a clearly distinct phenotype, with significantly increased number of blood vessels per field (p<0.05, Figure 2E).

### sst5TMD4 correlates with expression of angiogenic markers, lymphatic metastasis and disease-free survival in breast cancer patients

**Figure 3:**
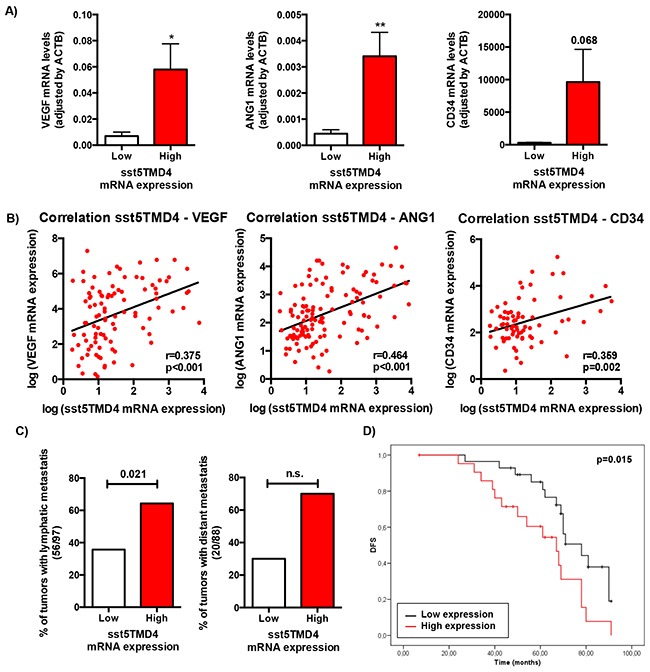
sst5TMD4 expression is associated to higher expression of pro-angiogenic factors and the presence of lymphatic metastasis in breast cancer samples **A.** Expression levels of VEGF, ANG1 and CD34 according to sst5TMD4 expression in the battery of 117 grade 3 infiltrating ductal breast carcinoma samples. Data represent mean ± SEM. **B.** Correlation between sst5TMD4 expression and the expression of VEGF, ANG1 and CD34 in breast carcinoma samples. **C.** Association between the presence of sst5TMD4 and lymphatic and distant metastasis in breast carcinoma samples. Graphs, obtained from a frequency table, show the distribution of 117 grade 3 ductal breast carcinoma with low or high sst5TMD4 expression according to lymphatic and distant metastasis. **D.** Kaplan-Meier plots showing the association between increased sst5TMD4 and disease-free survival (DFS) in breast carcinoma series. Significant correlation was studied using a Chi-square and Long-rank-p-value methods. Asterisks (*, p<0.05; **, p<0.01; ***, p<0.001) indicate significant differences between samples with low and high sst5TMD4 expression.

In order to explore the putative clinical consequences of sst5TMD4 presence, expression of this truncated receptor was determined by qPCR in a battery of 117 grade 3 infiltrating ductal breast carcinoma tumors (IDC) tumors resected between 2003 and 2004, and patients were categorized as low- or high-sst5TMD4 expression levels according to the median sst5TMD4 expression, in order to further analyze the putative association between sst5TMD4 presence and angiogenic markers and clinical data (Figure 3). This analysis revealed a clear association between the expression levels of the truncated receptor sst5TMD4 and the expression of two of the most potent pro-angiogenic factors (VEGF and Ang1) and one of the most clinically-relevant angiogenic markers (CD34), as the group of high-sst5TMD4 expressing tumors presented higher average levels of VEGF (p<0.05), Ang1 (p<0,01) and a clear tendency also in CD34 (p=0.068) (Figure 3A). Additionally, as might be expected, sst5TMD4 expression was directly correlated with the expression of these angiogenic markers in the whole battery of breast cancer samples (Figure 3B), supporting a clear association between sst5TMD4 expression and the angiogenic process. Moreover, the presence of the truncated receptor sst5TMD4 was also linked to a greater probability to develop metastasis, in that a high proportion of the breast cancers that underwent lymphatic metastasis presented high sst5TMD4 expression [p=0.021], and a similar, non-significant tendency was found for distant metastasis [p=0.092] (Figure 3C and Supplementary Table S2). More importantly, sst5TMD4 expression was also associated to disease-free survival in breast cancer patients, since patients with high sst5TMD4 expression exhibited a markedly lower disease-free survival (p=0.015) (Figure 3D).

**Figure 4:**
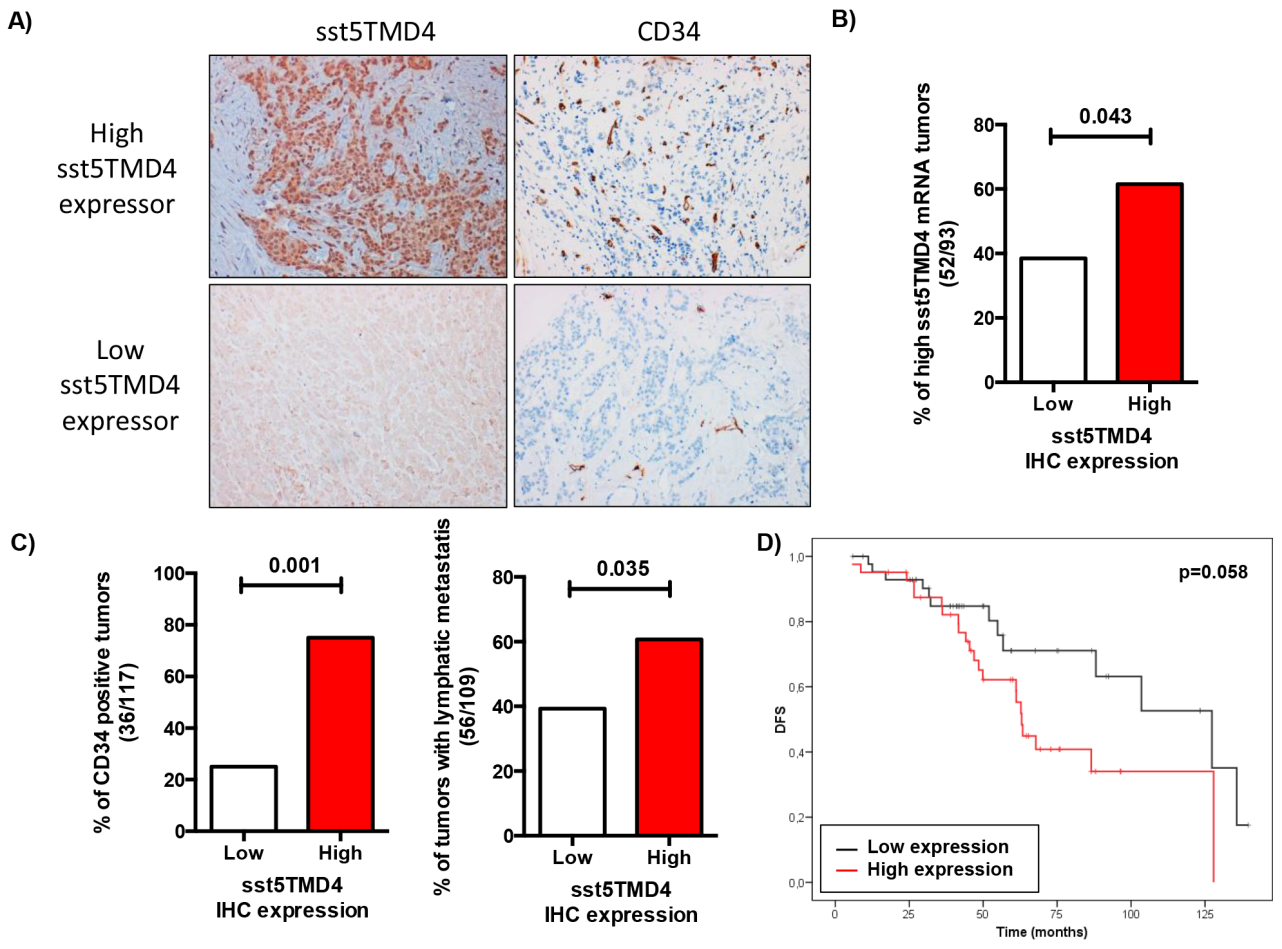
sst5TMD4 protein levels are associated to higher expression of pro-angiogenic factors and the presence of lymphatic metastasis in breast cancer samples **A.** A TMA including the 117 breast carcinoma samples was employed to determine the presence of sst5TMD4 at the protein level by using an sst5TMD4 specific custom-made antibody and of the angiogenic marker CD34. Representative pictures (x20) of sst5TMD4 and CD34 staining in low and high sst5TMD4 expressors are depicted. **B.** Association between presence and expression of sst5TMD4 at protein and mRNA levels in the battery of breast carcinoma samples. **C.** Association between the presence of sst5TMD4 and CD34 and lymphatic metastasis in breast carcinoma samples. Graphs, obtained from a frequency table, show the distribution of 117 grade 3 breast carcinoma with low or high sst5TMD4 protein levels according to CD34 staining and lymphatic metastasis. **D.** Kaplan-Meier plots showing the association of increased sst5TMD4 by IHC and disease-free survival (DFS) in breast carcinoma series. Significant correlation was studied using a Chi-square and Long-rank-p-value methods.

To further validate the association of truncated sst5TMD4 receptor with breast cancer aggressiveness, sst5TMD4 presence by immunohistochemistry was determined in these breast cancer samples using a TMA as described in Material and Methods. Representative images of some samples are shown in Figure 4A. Presence and expression of the sst5TMD4 receptor at the mRNA and protein levels were comparable [p=0.043] (Figure 4B and Supplementary Table S2). Interestingly, higher expression of sst5TMD4 at the protein level was also associated with CD34 positive tumors (p<0.001), with lymphatic metastasis (p=0.035) and with disease-free survival (p=0.058) (Figure 4C and Supplementary Table S2).

## DISCUSSION

Malignant tumors are characterized by the clonal expansion of genetically abnormal cells exhibiting uncontrolled proliferation, failure to respond to homeostatic signals, invasion of adjacent tissues, and metastasis [3]. Angiogenesis plays a rate-limiting role in tumor growth since, in the absence of angiogenesis, tumors display a ‘dormant phenotype’, where cell proliferation reaches equilibrium with death [16]. Under certain conditions, tumor cells can secrete excessive proangiogenic factors that stimulate the sprouting of new vessels [17]. When the angiogenic process is turned on, the tumor subsequently grows and becomes more likely to metastasize [3]. Multiple growth factor pathways participate in this intricate process to regulate the growth and maintenance of blood vessels; among them, VEGF, EGF and angiopoietins (Ang1, Ang2) are particularly important [18–20]. Hence, angiogenesis is a critical process for tumor growth and invasion and has become a promising target in cancer therapy.

Somatostatin and its analogues (SSAs) have been shown to tightly control the angiogenic process. Indeed, SSAs were reported to reduce vascular cell proliferation [21] and to prevent hypoxia-induced changes in VEGF/VEGFRs system in vascular cells [22], likely through the sst1 and/or sst4 receptors [22]. In addition, somatostatin and SSAs inhibit the angiogenic process in several models of retinal angiogenesis, likely acting through the sst2 receptor [23, 24]. Thus, and although much less is known about their role in tumoral cells, it seems that somatostatin and SSAs can reduce VEGF production from some types of tumoral cells such as gliomas [25], gastric carcinomas [26] or pancreatic cancer [27–29], acting through the sst2 receptor subtype [27–29]. Consistent with a role of somatostatin and its receptors in reducing the angiogenic process by acting at both, the endothelial cells and the tumoral cells levels [30], the use of SSAs in clinical trials has revealed that somatostatin could exert its anti-vasculogenesis effect by downregulating the serum VEGFs and, therefore, can be used as an important adjuvant to improve the survival of gastric cancer patients [26].

However, despite the fact that somatostatin receptors are densely expressed in breast cancer samples compared with normal tissues [31], being the sst2 subtype the most frequently and abundantly expressed in tumor cells [32], the clinical studies reporting treatment of breast cancer patients with SSAs have only demonstrated a limited success [33]. In this scenario, we have recently demonstrated that the presence of the truncated receptor sst5TMD4 correlates with a worse prognosis in a group of breast cancer tumors, and its overexpression is associated with increased malignant features such as invasion and proliferation abilities (both in cell cultures and nude mice) in breast cancer cell lines MCF-7 [10]. This was likely mediated by sst5TMD4-induced increase in phosphorylated ERK1/2 and Akt levels, which also led to mesenchymal-like phenotype. At the same time, this study demonstrated that sst5TMD4 interacts (physically and functionally) with sst2, promoting the disruption of somatostatin/sst2 inhibitory feedback. In the present study, we demonstrate that the presence of the sst5TMD4 [6–12] induces significant changes in the expression of several angiogenesis-related genes and increases the expression and/or secretion of pro-angiogenic factors such as VEGF, EGF and/or angiopoietins in the breast cancer cell lines MCF-7 and MDA-MB-231, where it also increases the capacity to form mammospheres in culture. Moreover, sst5TMD4 induces the expression of relevant pro-angiogenic factors in a breast cancer xenografts model derived from this cell line, which translated into an increased number of blood vessels in the tumors. Of note, our previous results provide a plausible basis for the findings shown herein in that sst5TMD4 could be inducing VEGF expression/secretion through a direct (increasing phosphorylated ERK and Akt levels [34]) and/or an indirect mechanism (disrupting the inhibitory loop established between somatostatin and the sst2 [22, 23, 28, 29]). Interestingly, the expression array indicated that the changes in VEGF expression/secretion were accompanied by changes in the expression of some of its receptors (i.e. NRP2), which could indicate the existence of an autocrine/paracrine loop in sst5TMD4-overexpressing cells; but, surprisingly, were not accompanied by changes in the expression of HIF-1a and HIF-1b, which suggest that these factors are more likely regulated at the protein level (amount and/or phosphorylation status) or that sst5TMD4 increases VEGF expression through an HIF-independent mechanism [35].

More importantly, the stimulatory role of sst5TMD4 on the production of pro-angiogenic factors from MCF-7 cells was accompanied by functional alterations in MCF-7 induced tumor xenografts. In particular, we have previously reported that sst5TMD4 overexpressing MCF-7 cells induce the formation of bigger xenograph tumors with a more undifferentiated phenotype [10]. In the present study, we expand those previous observations demonstrating that the presence of the sst5TMD4 receptor is associated with elevated production of pro-angiogenic factors (VEGF or EGF). Although we cannot rule out the possibility that other cell types present in the tumor could be contributing to these elevated levels of pro-angiogenic factors, the fact is that sst5TMD4-overexpressing cells induce tumors with higher number of blood vessels in the tumor, supporting its relevant role in tumoral angiogenic processes.

Angiogenesis is a crucial step for tumor growth and metastasis; however, tumor progression towards metastasis is a complex, multistage process, which is classically simplified as local invasion, intravasation, survival in the circulation, extravasation, and colonization [36]. Notwithstanding this, only about 0.2% of the tumor cells can effectively induce angiogenesis and eventually form metastases in distant organs [37]. In this context, cancer stem cells (CSCs) have been proposed to be the fundamental driving force of tumor development, initiation of invasion and metastasis [38]. Interestingly, as pointed earlier, the presence of the truncated sst5TMD4 receptors could induce an enrichment of the CSC-like population, as suggested by the enhanced capacity of sst5TMD4-expressing cells to form mammospheres *in vitro*, which supports that sst5TMD4 would be involved in several processes required for tumor progression, invasion, and, ultimately, metastasis. As such, sst5TMD4 has been found to be expressed at moderate or high levels in a representative proportion (approximately 40%) of samples from a cohort of 117 grade 3 IDC, which is consistent with that previously published wherein sst5TMD4 was detected in 28% of 49 breast cancer samples from a closed random series of tumor breast samples classified as poorly differentiated grade 3 (G3) tumors [10]. In this new series of human IDC samples, sst5TMD4 presence/expression is associated to a higher expression of several angiogenic markers (VEGF, ANG1 or CD34) and to an elevated capacity of the tumors to metastasize, mainly to lymph nodes, suggesting that sst5TMD4 enhances the risk of lymphatic metastasis. And, most importantly, sst5TMD4 presence/expression is associated with lower disease-free survival of patients, clearly reinforcing the notion that sst5TMD4 is involved in breast cancer malignancy and progression.

Thus, these results demonstrate that the mere presence of the truncated receptor sst5TMD4, which is overexpressed in a subset of breast cancer samples (herein and [10]), can directly or indirectly contribute to increase the capacity of the breast cancer cells to produce pro-angiogenic factors, thereby promoting the sprouting of new vessels, and facilitating tumor growth, which, in turn, makes them more prone to invade and metastasize. Consequently, when taken together, these and our previous discoveries support the notion that the presence/expression of the truncated receptor sst5TMD4 should be considered a risk factor for breast cancer progression, and therefore, that it could be used as a tool to identify novel, potentially valuable molecular biomarkers to improve the diagnosis and prognosis prediction for breast cancer, and as target to develop new drug therapies in these tumors.

## MATERIALS AND METHODS

### Breast cancer samples

The present study was performed using a total of 117 infiltrating ductal breast carcinoma (IDC) tumors obtained from the archives of the Pathology Department of MD Anderson Cancer Center, Madrid (Spain) and initially described at *Moreno-Bueno et al.* [39]. All tumors were grade 3. Patients underwent surgery between 2003 and 2004. The mean patient age at surgery was 54.9 years (range, 27 to 79 years). According to the TNM Classification staging, 48 of the tumors were stage I, 34 were stage II, and 35 were stage III-IV. Two different tumor areas from each sample were included into a tissue microarray (TMA) according to manufacturer's procedures. Histological and immunohistochemical studies were all carried out on formalin-fixed, paraffin-embedded tissue samples. Clinical data of the tumor sample are provided in Supplementary Table S3. This study was performed following standard ethical procedures of the Spanish regulation (Ley de Investigación Orgánica Biomédica, 14 July 2007) and was approved by the ethic committee of MD Anderson Cancer Center, Madrid, Spain.

### Cell lines

MCF-7 and MDA-MB-231 breast cancer cell lines (ATCC, Barcelona, Spain) were validated by analysis of STRs (GenePrint® 10 System, Promega, Barcelona, Spain) and checked for mycoplasma contamination by PCR as previously reported [40]. Cells were maintained in Dulbecco's Modified Eagle Medium (Sigma, San Louis, MO) supplemented with 10% fetal bovine serum, 1% antibiotic-antimycotic and 2mM L-glutamine, in a constant atmosphere with 37°C and 5% CO_2_. sst5TMD4 stably-transfected cells and their respective mock control (empty pCDNA3.1 plasmid transfected cells) have been previously generated and validated [10].

### Breast cancer xenografts model

Animal maintenance and experiments were carried out following the European Regulation for Animal Care under the approval of the University of Cordoba Research Ethics Committee. Six-week-old female athymic Swiss nu/nu mice were subcutaneously grafted in the flank with 2×10^6^ mock- or sst5TMD4-stably transfected MCF-7 cells (n=4-5 mice per condition). Tumor growth was monitored weekly during 3 months. Each tumor was dissected and different pieces were snap-frozen for qPCR analysis or formalin-fixed and sectioned for histopathological examination after hematoxylin-eosin staining as previously described [10].

### RNA extraction, reverse transcription and qPCR

RNA extraction, quantification, reverse-transcription as well as the development, validation and application of qPCR to measure the expression levels of different human transcripts have been previously reported elsewhere by our group [41–45]. Total RNA was extracted from paraffin-embedded breast cancer samples, frozen xenografted tumors and cell lines using Trizol (Life Technologies, Barcelona, Spain) following the manufacturer's protocol and subsequently treated with DNase (Promega, Barcelona, Spain). Total RNA concentration and purity was assessed using Nanodrop 2000 spectrophotometer (Thermo Scientific, Wilmington, NC, USA), and subsequently retro-transcribed using random hexamer primers and cDNA First Strand Synthesis kit (MRI Fermentas, Hanover, MD, USA). Complementary DNA derived from cell lines, xenografted tumors and breast cancer samples were amplified by quantitative real-time PCR (qPCR), where samples were run, in the same plate, against a standard curve to estimate mRNA copy number (1, 10^1^, 10^2^, 10^3^, 10^4^, 10^5^, and 10^6^ copies of synthetic cDNA template for each transcript) and a No-RT sample as a negative control. qPCR was performed using Brilliant II SYBR Green Master Mix in the Stratagene Mx3000p instrument (both from Agilent, La Jolla, CA, USA) as previously described [42–44]. Thermal profile consisted of a initial step at 95°C for 10 minutes, followed by 40 cycles of denaturation (95°C for 30 seconds), annealing (61°C for 1 minute), and extension (72°C for 30 seconds); and finally, a dissociation cycle to verify that only one product was amplified. To control for variations in the amount of RNA used and the efficiency of the reverse-transcription reaction and, the expression level (copy number) of each transcript was adjusted by ACTB expression levels. It should be noted that, as previously reported [43, 44] and based on the stringent criteria to maximize specificity and efficiency, the qPCR technique, as applied, can be used to accurately quantify copy numbers for all human transcripts included in this study (see list of primers used in Supplementary Table S4).

### Microarray gene expression profile

Microarray experiments were performed using Human Whole Genome array V2 4*44K array G4845A (Agilent Technologies, Santa Clara, CA, USA). Four independent passages from stably-transfected sst5TMD4-pCDNA3.1 and empty-pCDNA3.1 vector, used as control (mock), MFC7 cells were used. Total RNA from cell lines was isolated using Trizol reagent (Life Technologies) as indicated by the manufacturer. Purity of isolated RNA was evaluated spectrophotometrically by the A260/A280 absorbance ratio. RNA was labeled and array hybridized using the Low RNA Linear Amplification Kit and the In Situ Hybridization Kit Plus (Agilent technologies), respectively, following manufacturer's protocol. After hybridization and washing, the slides were scanned in an Axon GenePix Scanner (Axon Instruments) and analyzed using Feature Extraction Software 10.0 (Agilent technologies). RNA samples from independent sst5TMD4-stably transfected cells were labeled with Cy5-dUTP and equal concentration of each RNA from mock control cells were labeled with Cy3-dUTP. Differentially expressed genes in sst5TMD4-transfected MCF-7 cells vs. control (mock) cells were identified with GEPAS (Gene Expression Pattern Analysis Suite, http://gepas3.bioinfo.cipf.es) selecting genes with a fold difference of at least two in all of the samples and standard deviation lower than 0,5. Functional enrichment analysis was performed using the FatiGO application (http://babelomics.bioinfo.cipf.es). Microarray raw data tables have been deposited in the Gene Expression Omnibus.

### VEGF determination by ELISA

VEGF concentration in the sst5TMD4- and mock-transfected MCF-7 culture media was determined using a commercial human VEGF ELISA kit (VEGF human ELISA kit; Cat. number: KHG0112; Life Technologies), following the manufacturer's instructions. The information regarding specificity, detectability and reproducibility for the assay can be accessed at the company website.

### Mammosphere formation assay

Mammosphere formation assays were developed following previously published protocols [46]. Briefly, 10.000 sst5TMD4- and mock-transfected MCF-7 or MDA-MB-231 cells were seeded (in triplicate) in 6-well plates pretreated with Poly(2-hydroxyethyl methacrylate) in 2ml DMEM/F12 without phenol red. Culture media was supplemented with recombinant epithelial growth factor, B27 supplement and Gentamicin/Amphotericin B. After seven days of incubation at 37°C, 5%CO2, the number of mammospheres was counted using an inverted microscope.

### Western blotting

Xenografted tumors were processed for the detection of sst5TMD4 and VEGF by western blot using standard procedures [10]. Specifically, proteins were extracted from a tumoral piece using pre-warmed SDS-DTT sample buffer (62,5mM Tris-HCl, 2% SDS, 20% glicerol, 100mM DTT and 0,005% bromophenol blue) followed by sonication for 10 sec and boiling for 5 min at 95°C. Proteins were separated by SDS-PAGE and transferred to nitrocellulose membranes (Millipore). Membranes were blocked with 5% non-fat dry milk in Tris-buffered saline/0.05% Tween 20 and incubated with the rabbit polyclonal antisera against human sst5TMD4 previously described [6, 10] or the specific goat anti-human VEGF antibody (AB-293-NA; R&D systems, Minneapolis, MN, USA) and the appropriate secondary antibodies (anti-rabbit or anti-goat IgG-HRP; Santa Cruz Biotechnology, Dallas, TX, USA). Proteins were developed using an enhanced chemiluminescence detection system (GE Healthcare, Madrid, Spain) with dyed molecular weight markers. A densitometric analysis of the bands was carried out with ImageJ software [47] using total protein loading (Ponceau staining) as normalizing factor as previously reported elsewere [48].

### Immunohistochemical and immunohistofluorescence analysis

Xenografted tumors were processed for the detection of VEGF by immunohistofluorescence using standard procedures [10]. Specifically, a tumoral piece was formalin-fixed and paraffin-embedded. After antigen-retrieval, sections were incubated with a specific anti-human VEGF antibody (AB-293-NA; R&D systems) and the appropriate fluorescence-labeled secondary antibody (Donkey Anti-Goat IgG Alexa Fluor 488; ab150129; Abcam, Cambridge, UK). Signal intensity was determined using the ImageJ software [47].

sst5TMD4 and CD34 immunohistochemical staining of the human breast cancer samples was performed by the LSAB (Dako) method with a heat-induced antigen retrieval step. Briefly, sections were immersed in boiling 10mM sodium citrate at pH 6.0 for 3 min in a pressure cooker. Rabbit polyclonal antisera against human sst5TMD4 previously described [6, 10] and monoclonal mouse Anti-Human CD34 Class II (Clone QBEnd 10, Dako) were used as primary antibodies, and goat anti-rabbit (ThermoScientific, Barcelona, Spain) and rabbit anti-mouse (ThermoScientific) as secondary antibodies. The antibodies were used at 1:1000 dilutions. The primary antibodies were omitted in the negative controls. sst5TMD4 staining were categorized as low or high expression refers to the normal sst5TMD4 staining. CD34 staining was categorized as low or high with respect to normal mammary tissue.

### Determination of blood vessels in the xenografted tumors

Xenografted tumors were processed for the quantification of blood vessel density using a standard histopathological procedure [49]. Specifically, a tumor piece was formalin-fixed and paraffin-embedded followed by haematoxylin and eosin staining. Vascular density was determined by counting the number of visible vessels per 20x objective tumor-full fields from a minimum of 5 fields per each of the 4-5 animals per genotype.

### Statistical analysis

For *in vitro* experiments, data are expressed as mean ± SEM obtained from, at least, three separate, independent experiments carried out in different days and with different cell preparations. Statistical analysis was carried out using Student's t-test. For *in vivo* xenografts experiments, data are expressed as mean ± SEM obtained from n=4-5 animals. Statistical analysis was carried out using Student's t-test. For the analysis of human breast carcinoma data, samples were categorized in low and high sst5TMD4 levels according to median sst5TMD4 expression levels. Differences in the expression of angiogenic markers between both groups were assessed by Student's t-test. Correlations between the expression of sst5TMD4 and angiogenic markers was assessed by Pearson's correlation test. Significant correlations between categorized sst5TMD4 mRNA/protein expression, CD34 IHC expression, presence of metastasis and disease-free survival were studied using Chi-square and Long-rank-p-value methods. Statistical analyses were carried out with GraphPad Prism 6 and SPSS 17.0 (IBM). P-values smaller than 0.05 were considered statistically significant.

## SUPPLEMENTARY FIGURE AND TABLES




