# Supplementary material for: The truncated somatostatin receptor sst5TMD4 stimulates the angiogenic process and is associated to lymphatic metastasis and disease-free survival in breast cancer patients
Source: Oncotarget. 2016 Aug 5;7(37):60110–22. doi: 10.18632/oncotarget.11076 (PMC5312372; doi:10.18632/oncotarget.11076)
Supplement: Supplementary file 1 [file oncotarget-07-60110-s001.pdf]

The truncated somatostatin receptor sst5TMD4 stimulates the angiogenic process and is associated to lymphatic metastasis and disease-free survival in breast cancer patients

SUPPLEMENTARY FIGURE AND TABLES

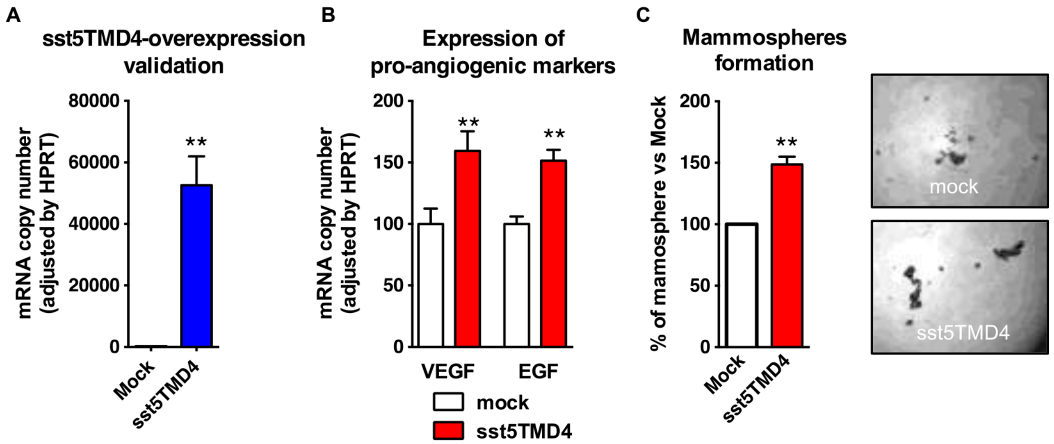

Supplementary Figure S1: sst5TMD4 expression is associated to higher expression of pro-angiogenic factors and higher capacity to form mammospheres in breast cancer MDA-MB-231 cells. **A.** Confirmation of sst5TMD4 overexpression in MDA-MB-231 by qPCR. **B.** Changes in the expression of pro-angiogenesis genes (VEGF and EGF) measured by qPCR in MDA-MB-231 cells stably transfected with sst5TMD4 or pCDNA3.1 empty vector (mock). **C.** Percentage and representative images of mammospheres formed from MDA-MB-231 cells stably transfected with ss5TMD4 and the respective mock controls. Data represent mean ± SEM of n=3-6 independent experiments. Asterisks (**, $p<0.001$) indicate significant differences between sst5TMD4- and mock-transfected MDA-MB-231 cells.

Supplementary Table S1: List of genes found to be altered in the gene expression microarray comparing sst5TMD4-transfected MCF-7 cells with mock-transfected MCF-7 cells.

See Supplementary File 1

Supplementary Table S2: Relationship between sst5TMD4 expression and clinico-pathological e immunohistochemical features in IDC grade 3 samples.

	sst5TMD4* mRNA expression: n (%)		
	**Low**	**High**	
**Lymph node metastasis (n=96)**			
Negative (n=41)	24 (58.5)	17 (32.1)	p=0.021
Positivie (n=5)	20 (41.5)	36 (67.9)	
**Distant metastasis (n=95)**			
Negative (n=68)	34 (85.0)	34 (70.8)	p=0.092
Positive(n=20)	6 (15.0)	14 (29.2)	
	**sst5TMD4 protein expression: n(%)***		
	**Low**	**High**	
***sst5TMD4 mRNA expression* (n=93)**			
Low (n=41)	24 (54.5)	17 (34.7)	p=0.043
High (n=52)	20 (45.5)	32 (65.3)	
**CD34 (n=117)**			
Low (n=81)	52 (85.2)	29 (51.8)	p<0.001
High (n=36)	9 (14.8)	27 (48.2)	
**Lymph node metastasis (n=109)**			
Negative (n=53)	31 (58.5)	22 (39.3)	p=0.035
Positive (n=56)	22 (41.5)	34 (60.7)	
**Distant Metastasis (n=111)**			
Negative (n=82)	40 (70.1)	42 (77.8)	N.S
Positive (n=29)	17 (29.9)	12 (22.2)	

*n(%), number of analysed cases and (percentage).
N.S: No statistical significance

Supplementary Table S3: Summary of clinical, pathological, immunohistochemical and molecular features of IDC Grade 3 samples

	n (%)*
**sst5TMD4 protein expression (n=117):**	
Low	61 (52.1)
High	56 (47.9)
sst5TMD4 mRNA expression **(n=98):**	
Low	44 (44.9)
High	54 (55.1)
**CD34 protein expression (n=117):**	
Low	81 (69.2)
High	36 (30.8)
**Lymph node metastasis (n=117):**	
Negative	56 (47.9)
Positive	61 (52.1)
**Distant metastasis (n=117):**	
Negative	82 (70.1)
Positive	35 (29.9)

*n(%), number of analysed cases and (percentage).

Supplementary Table S4: Primer sequences, product sizes and GeneBank accession numbers

Gene	Sense	Antisense	Product lenght (bp)	GeneBank Accession number
sst5TMD4	TACCTGCAACCGTCTGCC	AGCCTGGGCCTTTCTCCT	98	DQ448304
VEGFA	TTAAACGAACGTACTTGCAGATG	GAGAGATCTGGTTCCCGAAA	93	NM_001171623.1
EGF	CTGAAGGTACTCTCGCAGGAAA	CACTGAGACACCAGCATCCAC	146	NM_001963.4
ANGPT1	GACAGATGTTGAGACCCAGGTA	TCTCTAGCTTGTAGGTGGAT AATGAA	89	NM_001146.3
ANGPT2	GGATGGAGACAACGACAAATG	GGACCACATGCATCAAACC	78	NM_001147.2
CD34	CACCAATCTGACCTGAAAAAGC	AAATAGCCAGTGATGCCCAAG	143	NM_001025109.1
HIF1a	TTAGATTTTGGCAGCAACGAC	GGGTGAGGGGAGCATTACA	87	NM_001530.3
HIF1b	ACTACTGCCAACCCCGAAAT	ATGGCTCCTCCACCTTGAAT	98	NM_001668.3
IGFBP1	GTTTAGCCAAGGCACAGGAG	TATCTGGCAGTTGGGGTCTC	203	NM_000596.2
ITGB2	ACTGATGACGGCTTCCATTT	GATGGGCTGGATGTTGTTTT	171	NM_000211.4
MMP1	CTGATATCGGGGCTTTGATG	GATGGGCTGGACAGGATTTT	122	NM_002421.3
MMP10	TCGCAAGATGATGTGAATGG	TGATGGCATCGAAGGACAAA	145	NM_002425.2
CSF3	CCTCCCCATCCCATGTATTT	TGGGAGGACAGGAGCTTTTT	167	NM_000759.3
18S	CCCATTCGAACGTCTGCCCTATC	TGCTGCCTTCCTTGGATGTGGTA	136	NR_003286.2
ACTB	ACTCTTCCAGCCTTCCTTCCT	CAGTGATCTCCTTCTGCATCCT	176	NM_001101
