# Supplementary material for: The truncated somatostatin receptor sst5TMD4 stimulates the angiogenic process and is associated to lymphatic metastasis and disease-free survival in breast cancer patients
Source: Oncotarget. 2016 Aug 5;7(37):60110–22. doi: 10.18632/oncotarget.11076 (PMC5312372; doi:10.18632/oncotarget.11076)
Supplement: Supplementary file 2 [file oncotarget-07-60110-s002.docx]

| **Supplemental Table 1**. List of genes found to be altered in the gene expression microarray comparing sst5TMD4-transfected MCF-7 cells with mock-transfected MCF-7 cells | | | | |
| --- | --- | --- | --- | --- |
|  |  |  |  |  |
| GenBank Accession | Fold change | Gene Name | Function | UniGene |
| **NM_002421** | **-6.12** | **MMP1** | **EMT related** | **Hs.83169** |
| NM_001999 | -5.50 | FBN2 | EMT related | Hs.519294 |
| **NM_002425** | **-4.31** | **MMP10** | **EMT related** | **Hs.2258** |
| NM_033056 | 2.11 | PCDH15 | EMT related | Hs.672170 |
| NM_007074 | 4.62 | CORO1A | EMT related | Hs.415067 |
| **NM_000211** | **5.30** | **ITGB2** | **EMT related** | **Hs.375957** |
| **NM_018934** | **6.36** | **PCDHB14** | **EMT related** | **Hs.658497** |
| **NM_000873** | **7.37** | **ICAM2** | **EMT related** | **Hs.431460** |
| **NM_002543** | **-36.62** | **OLR1** | **angiogenesis/inflammation related** | **Hs.412484** |
| **NM_017852** | **-15.04** | **NLRP2** | **angiogenesis/inflammation related** | **Hs.369279** |
| **NM_000759** | **-5.31** | **CSF3** | **angiogenesis/inflammation related** | **Hs.2233** |
| **NM_181780** | **-2.97** | **BTLA** | **angiogenesis/inflammation related** | **Hs.445162** |
| **NM_001768** | **-2.66** | **CD8A** | **angiogenesis/inflammation related** | **Hs.85258** |
| **NM_007268** | **-2.01** | **VSIG4** | **angiogenesis/inflammation related** | **Hs.8904** |
| **NM_176810** | **2.15** | **NLRP13** | **angiogenesis/inflammation related** | **Hs.446924** |
| **NM_080600** | **4.05** | **CD22** | **angiogenesis/inflammation related** | **Hs.643440** |
| **NM_201264** | **4.78** | **NRP2** | **angiogenesis/inflammation related** | **Hs.471200** |
| **NM_201266** | **5.39** | **NRP2** | **angiogenesis/inflammation related** | **Hs.471200** |
| **NM_001048** | **-4.03** | **SST** | **cell growth** | **Hs.12409** |
| NM_002193 | -3.85 | INHBB | cell growth | Hs.1735 |
| NM_021020 | -2.92 | LZTS1 | cell growth | Hs.521432 |
| BC037837 | 2.29 | RBM45 | cell growth | Hs.377257 |
| NM_000596 | 9.06 | IGFBP1 | cell growth | Hs.642938 |
| BC002763 | -5.25 | PRKAR2A | signal tranduction | Hs.631923 |
| **NM_012242** | **-3.46** | **DKK1** | **signal tranduction** | **Hs.40499** |
| **AI093683** | **-3.22** | **GPRC5B** | **signal tranduction** | **Hs.148685** |
| **AK096323** | **-2.82** | **SGPP2** | **signal tranduction** | **Hs.591604** |
| **NM_024866** | **-2.53** | **ADM2** | **signal tranduction** | **Hs.647465** |
| **NM_001715** | **-2.23** | **BLK** | **signal tranduction** | **Hs.146591** |
| NM_001006627 | 2.01 | CHRM2 | signal tranduction | Hs.535891 |
| **BX648591** | **3.78** | **CNTN1** | **signal tranduction** | **Hs.143434** |
| **NM_020168** | **3.86** | **PAK6** | **signal tranduction** | **Hs.513645** |
| NM_001740 | 4.85 | CALB2 | signal tranduction | Hs.106857 |
| NM_014365 | -4.36 | HSPB8 | basic cellular function | Hs.400095 |
| NM_178012 | -3.19 | TUBB2B | basic cellular function | Hs.300701 |
| **NM_022844** | **-2.37** | **MYH11** | **basic cellular function** | **Hs.460109** |
| AK125361 | 5.91 | MGAT3 | basic cellular function | Hs.276808 |
| NR_000041 | 6.96 | RNU12 | basic cellular function | Hs.511989 |
| **NM_001018036** | **-2.34** | **TSHR** | **Metabolism** | **Hs.160411** |
| AL832067 | -2.01 | MDH1 | Metabolism | Hs.526521 |
| NM_003578 | 2.74 | SOAT2 | Metabolism | Hs.656544 |
| NM_016327 | 3.18 | UPB1 | Metabolism | Hs.474388 |
| NM_004668 | 3.29 | MGAM | Metabolism | Hs.122785 |
| NM_001001711 | -2.19 | DDI1 | DNA damage | Hs.591941 |
| **NM_004083** | **-2.11** | **DDIT3** | **DNA damage** | **Hs.505777** |
| NM_017638 | -6.94 | MED18 | Trasncription related | Hs.479911 |
| AL832834 | -5.29 | ZNF568 | Trasncription related | Hs.404220 |
| NM_017831 | -4.41 | RNF125 | Trasncription related | Hs.633703 |
| NM_024967 | -2.81 | ZNF556 | Transcription related | Hs.287433 |
| **BX344068** | **2.13** | **TXNIP** | **Transcription related** | **Hs.702728** |
| **NM_004827** | **-7.31** | **ABCG2** | **Transport** | **Hs.480218** |
| NM_152908 | 3.32 | SLC47A2 | Transport | Hs.126830 |
| NM_001017920 | 4.67 | DAPL1 | cell death related | Hs.59761 |
| NM_032526 | -2.19 | NT5C1A | Hypoxia related | Hs.307006 |
| NM_005025 | -2.88 | SERPINI1 | cell reorganization | Hs.478153 |
| AK026387 | -15.92 | LOC649898 | miscellaneous | Hs.306842 |
| BC017970 | -9.02 | FLJ44894 | miscellaneous | Hs.631635 |
| NM_153022 | -6.34 | C12orf59 | miscellaneous | Hs.226422 |
| BQ716254 | -5.89 | BQ716254 | miscellaneous | Hs.596087 |
| BQ003493 | -4.50 | BQ003493 | miscellaneous | Hs.665412 |
| XR_018087 | -4.38 | LOC347097 | miscellaneous | Hs.586453 |
| BE004814 | -3.03 | BE004814 | miscellaneous |  |
| XM_292820 | -2.94 | LOC342979 | miscellaneous | Hs.444298 |
| AK097805 | -2.85 | AK097805 | miscellaneous | Hs.661772 |
| AK124080 | -2.46 | AK124080 | miscellaneous | Hs.446041 |
| AK093303 | -2.21 | AK093303 | miscellaneous | Hs.511522 |
| AK000832 | -2.19 | AK000832 | miscellaneous | Hs.610967 |
| NP283818 | -2.15 | NP283818 | miscellaneous |  |
| AI241561 | -2.01 | AI241561 | miscellaneous |  |
| AL109704 | -2.01 | AL109704 | miscellaneous | Hs.651358 |
| AY358788 | 2.22 | AY358788 | miscellaneous | Hs.702753 |
| AK023259 | 2.23 | FLJ13197 | miscellaneous | Hs.29725 |
| AF318333 | 2.56 | AF318333 | miscellaneous | Hs.684469 |
| AK096685 | 3.94 | AK096685 | miscellaneous | Hs.658287 |
| NM_032823 | 4.81 | C9orf3 | miscellaneous | Hs.434253 |
| NM_001002919 | 8.60 | MCG_1990170 | miscellaneous | Hs.355207 |
| AK024680 | 13.40 | AK024680 | miscellaneous | Hs.660596 |
| NM_182511 | -6.14 | CBLN2 | unkown function | Hs.569851 |
| NM_006393 | -4.67 | NEBL | unkown function | Hs.5025 |
| **NM_014729** | **-4.32** | **TOX** | **unkown function** | **Hs.491805** |
| NM_030901 | -2.01 | OR7A17 | unkown function | Hs.247717 |
| **NM_022450** | **2.41** | **RHBDF1** | **unkown function** | **Hs.57988** |
| NM_032563 | 4.09 | LCE3D | unkown function | Hs.244349 |
| NM_006228 | 4.75 | PNOC | unkown function | Hs.88218 |
| NM_052816 | 5.13 | TRIM31 | unkown function |  |
| NM_173689 | 5.63 | CRB2 | unkown function | Hs.568340 |
| Genes found to be associated to angiogenic process after the supervised user-driven functional analysis are shown in bold | | | | |
